# Medial humeral epicondylar lesions and discreet calcified structures in the canine elbow: radiographic description of 183 cases

**DOI:** 10.1186/s13028-020-00556-w

**Published:** 2020-10-20

**Authors:** Arne Magnus Rørvik, Cathrine Trangerud, Jorunn Grondalen

**Affiliations:** Department of Companion Animal Clinical Sciences, Faculty of Veterinary Medicine, Norwegian University of Life Sciences, P.O. Box 369, N-0102 Centrum, Oslo Norway

**Keywords:** Dog, Elbow, Flexor enthesopathy, Medial humeral epicondyle

## Abstract

**Background:**

In this 4-year prospective observational study, all elbows in a dysplasia screening program including 14,073 dogs were studied using radiographs in two projections. Elbows were evaluated for the presence of medial humeral epicondylar lesions or discreet calcified structures and were described as they appeared. The age, breed, and sex of affected dogs were recorded. The prevalence for each lesion was calculated exclusively on breeds where the number of radiographed dogs exceeded 500.

**Results:**

Medial humeral epicondylar lesions or medial discreet calcified structures were diagnosed in 183 dogs and 211 elbows. The prevalence of true Flexor enthesopathy (FE) in this Norwegian population of mainly young, large breed dogs was calculated to be approximately 1.4 per 1000 dogs and varied by breed. Also, the prevalence of the other lesions varied considerably by breed. The most common finding was discreet calcified structures, termed medial ossified structures (MOS) (0.7%).

In elbows affected with fragmented medial epicondyles (FME) (0.07%) and especially FE (0.14%), the degree of periarticular new bone formation (PNBF) was increased when compared to unaffected elbows. In joints affected with MOSs or medial lucent lesions MLLs (0.25%), there was no difference in the presence or degree of PNBF compared to unaffected joints, even in older dogs.

**Conclusions:**

The prevalence of medial humeral epicondylar lesions and MOSs differs considerably among dog breeds. Elbow joints with FMEs and particularly FE had a highly increased presence and degree of PNBF compared to joints without these lesions. Elbow joints with MOSs or MLLs did not have an increased presence or degree of PNBF compared to joints without these lesions.

## Background

While scrutinizing canine elbows in the Norwegian elbow dysplasia (ED) screening program for several years, we came across various forms of lesions of the medial epicondyle or medial discreet calcified structures, which were poorly described in the literature at that time. The ED screening program was intended to change the method of screening using only flexed mediolateral (M-L) views in grading instead of two projections. It was decided to initiate a prospective observational study on the observed lesions before the craniocaudal (Cr-Cd) projection was no longer available in the screening program.

Early studies on these lesions were reports of a few lame dogs where a calcified object was surgically removed [1–7]. In a study, the authors described a lesion on a radiograph as a ‘partial ununited epicondyle’, but called the lesion ‘ununited medial epicondyle’ (UME) and presented it as a developmental lesion and a new form of ED [5]. It was opposed in another report, stating that neither three similar cases presented, nor the one previously presented, were UME [6]. It was postulated that UMEs do not actually occur and that the lesion was rather a fragmentation of the medial epicondyle. May and Bennet [7] reported four cases of flexor enthesopathy (FE) and were the first to describe the clinical and radiological findings of these lesions.

In later studies, the number of cases increased [8–13]. Forty cases of what was called ‘medial epicondylar lesions’ were separated into two groups of 20; one group with evidence of FE and one with discreet mineralised fragments [9]. Few reports have described the histology of these structures. Meyer-Lindenberg et al. [8] described the histology of 13 structures that had been surgically removed from the joints when they reported the findings of 23 cases of ‘metaplasia’, and they reported that five structures contained pure bone.

What was called ‘ununited medial epicondyles’ was found in 15% of the puppies in a follow-up study of seven litters of Labrador Retrievers (46 dogs). The degree of osteoarthritis and clinical signs were equally expressed in the affected and non-effected elbows during the whole observation time, ending at the age of 6–8 years. The authors stated that the term ‘UME’ was not appropriate for the described lesions and that the lesions in these elbows had no clinical impact. They also suggested that these lesions were more frequently present in dogs than previously described [14].

In a review on medial humeral epicondylar lesions, the authors frequently found that calcified bodies near the medial epicondyle were described using several terms, including ‘UME’. They concluded that this expression was unsuited and proposed that the term ‘flexor enthesopathy’ should be used instead to describe the presence of pathological changes within the flexor muscles and their attachment to the medial epicondyle, whatever the cause of the actual problem [15].

In a study of 117 dogs with elbow lameness, radiographic findings in the medial humeral epicondyle were studied. Diagnosis was confirmed either by computed tomography, magnetic resonance imaging, or arthroscopy depending on the type of elbow problem. Eighty elbows were recorded with lesions in the medial humeral epicondyle. In 12 of these radiographic changes of the medial epicondyle were the only findings and these were diagnosed with ‘primary flexor enthesopathy’. The 68 other elbows with medial epicondylar lesions had other major lesions present, mostly medial coronoid disease (MCD), and the medial epicondylar lesions in these elbows were called ‘concomitant flexor enthesopathy’. The authors made a very distinct separation between elbows with specific changes in the enthesis of the medial epicondyle itself (medial epicondylar lesions) and elbows with normal medial epicondyles. All elbows with medial epicondylar lesions (80 elbows with FE, primary or concomitant) had irregular bone structure on the distal edge of the medial epicondyle and/or ‘bony spurs’ distal-caudally, except for two elbows that only had an irregular bone structure [10].

In another study, 13 elbows (8 dogs) were diagnosed with primary FE. These elbows had low-grade or no osteoarthritis and no calcification in the soft tissue. Clinical examination, radiography, ultrasound, CT, MRI, arthroscopy, and open surgery were used. Specific pathological changes in the soft tissue and bone were described. All elbows had bony spurs and irregular bone structure distally in the epicondyle on radiography except in one dog. This dog had bilateral FE, but the only radiographic finding in the elbows except from low grade osteoarthritis was dense sclerosis around a distinct radiolucent area centrally in the medial epicondyle in both elbows [11].

Previous studies have predominantly included lame dogs collected during clinical examination and/or treatment. The aim of our study was to use a Norwegian population of dogs radiographed for ED, which is a large and defined population not significantly biased by clinical symptoms, and to describe radiographic variations of medial humeral epicondylar lesions and medial calcified structures and find the prevalence of these lesions in the population. This population seemed to represent the true population of young dogs, at least for some specific breeds in Norway. Our hypothesis was that there was no difference in prevalence between breeds.

Another aim was to evaluate the association between the lesions and periarticular new bone formation (PNBF) on a large scale. The presence of PNBF might indicate whether these lesions have a harmful impact on the joint, which is difficult to evaluate in individual joints when inflammation is present. Our hypothesis was that there was more PNBF in elbows with lesions.

Such a study is unique, but for various reasons the study was not published. When the interest for these lesions increased, we found that the results of this study might provide additional and important knowledge if the radiographs were re-interpreted considering the results of new knowledge.

## Methods

This was a prospective observational study. Elbow radiographs from the Norwegian ED screening program were studied during a four-year period. They were evaluated for the presence of medial humeral epicondylar lesions and discreet mineralised bodies in the soft tissue as they arrived. The radiographs were of both elbows in at least one flexed M-L and one Cr-Cd projection (mostly Cr15L-CdM-O). The elbows of 14,073 dogs were studied. Radiographs showing distinct calcified structures in the medial or mediocaudal aspects of the elbows or lesions in the humeral medial epicondyle itself were put aside for further studies. The study was conducted from 1998 to 2003 and the radiographs were re-interpreted in 2015.

The age, sex, and breed of the affected dogs were recorded. Data from the Norwegian Kennel Club (NKC) were retrieved. These data included the number of dogs radiographed for ED within each affected breed in the 4-year study period and the number of new registrations of these breeds in the year before the radiographic examination (1997–2002).

The various lesions were categorised into four groups in 2015: flexor enthesopathy (FE), fragmented medial epicondyle (FME), medial ossified structure (MOS), and medial lucent lesion (MLL).

FE was characterised by the presence of a bony spur formation on the distal-caudal end of the epicondyle and/or irregular bone structure in the distal border of the epicondyle itself.

An FME was characterised by a significant crater in the distal end of the medial epicondyle and one or more adjacent bony fragments caudomedially in the elbow.

An MOS was characterised by one or more discreet, mineral opaque, smoothly delineated objects medially in the elbow, where the medial epicondyle itself was essentially normal, with no bony spur formation on the distal-caudal end of the epicondyle and no irregular bone structure in the distal border of the epicondyle itself.

An MLL was characterised by a radiolucent area medially in the epicondylar bone with a consistent location close to the physis and a small ossified structure embedded in it. The medial humeral epicondyle was perfectly normal.

The locations of MOSs were recorded and the relative size of the mineralised structures in MOSs and MLLs were estimated by measuring the width and length of the radiographic shadow. The size of the lucent bone in an MLL varied but was not measured.

PNBF includes enthesophytes in the capsular attachment and/or osteophytes in the periphery of the articular surface and/or periosteal reaction near the capsular attachment [16–19]. PNBF was graded from 0 to 3, by evaluating the cranial border of the anconeal process, the cranial contour of the medial coronoid process, the caudal rim of the lateral epicondyle, the cranial contour of the humeral condyle, and the cranial proximal end of the radial head. The grade of PNBF, not the ED score, was used to compare secondary changes in the joints.

Elbows where PNBF exceeded the distal-caudal tip of the medial epicondyle in an excessive manner were excluded.

Statistical analyses were performed using JMP^®^ Pro 13.0.0 (SAS Institute; Cary, NC, USA). Calculations on occurrences were performed exclusively on breeds where the number of radiographed dogs exceeded 500. Pearson’s chi-squared test was used to evaluate the distribution of sex in each category. The degree of PNBF in affected and non-affected joints was compared using Pearson’s chi-squared test. In addition, a Pearson’s chi-squared test was used to evaluate if the presence of PNBF increased with age in elbows affected with an MOS. Two age groups were used: dogs 2 years of age or younger and dogs older than 2 years. The same age groups were used when a possible difference in the size of MOSs in younger and older dogs were evaluated using the Student’s t-test. In elbows with more than one ossified structure, the sum of the estimated sizes was used. Significance level was set at *P* < 0.05.

## Results

Of the 14,073 dogs studied, 183 dogs (1.3%) were diagnosed with medial humeral epicondylar lesions or discreet calcified structures in one or both elbows. Individuals of 28 breeds were affected, including three breeds of Belgian Shepherd. Both sexes were equally represented regarding these lesions. The number of affected dogs and elbows within the categories of lesions for each affected breed are presented in Table 1, together with the data retrieved from the NKC for each of these breeds.

**Table 1:** Number of dogs (elbows) with presence of medial epicondylar lesions and medial ossified structures in the elbows found in a 4-year prospective study in the Norwegian ED screening program

Breed	New reg. 97–02	No of radio-graphed dogs 98–03	MOS	MLL	FME	FE	Un-even bilat^a^	SUM
German Shepherd	11,059	3524	21 (21)	9 (10)	1 (1)		a 1 (2)	32 (34)
Labrador Retriever	3891	1813	24 (26)	9 (9)	2 (2)	6 (8)		41 (45)
Rottweiler	3460	1757	12 (14)	9 (10)	2 (2)	–	d 1 (2)	24 (28)
Bernese Mountain Dog	1507	1147	12 (12)	–	–	–		12 (12)
Golden Retriever	5696	702	4 (4)	1 (1)	1 (1)	–		6 (6)
Leonberger	1217	628	–	1 (1)	–	1 (2)		2 (3)
Newfoundland	1173	574	22 (29)	1 (1)	–	6 (7)	c 1 (2)	30 (39)
Belgian Shepherd	1653	584	2 (2)	2 (3)	1 (1)	-		5 (6)
Rhodesian Ridgeback	560	273	1 (1)	2 (4)	–	–		3 (5)
Flat Coated Retriever	3043	247	1 (1)	-	1 (1)	–		2 (2)
Chow Chow	403	144	–	–	–	3 (4)		3 (4)
Boxer	1657	111	2 (2)	–	–	–		2 (2)
Irish Wolfhound	301	110	1 (1)	–	–	–		1 (1)
Australian Shepherd	192	89	1 (2)	–	–	–		1 (2)
Bull Mastiff	231	87	2 (3)	–	–	1 (1)		3 (4)
Giant Schnauzer	870	82	1 (1)	–	–	–		1 (1)
Dobermann	1022	41	1 (1)	–	–	–		1 (1)
Howavart	187	39	1 (1)	–	–	–		1 (1)
Greater Swiss Mountain Dog	37	30	–	1 (1)	–	–		1 (1)
St. Bernard	223	30	–		–	3 (3)	b 1 (2)	4 (5)
Chesapeake Bay Retriever	66	26	–	–	1 (1)	–		1 (1)
English Springer Spaniel	1370	26	1(1)	1 (1)	–	–		2 (2)
English Setter	6070	25	–	–	1 (1)	–	d 1 (2)	2 (3)
Mastiff	147	24	1 (1)	–	–	–		1 (1)
German Wirehaired Pointer	1071	16	1 (1)	–	–	–		1 (1)
Curly Coated Retriever	55	4	1 (1)	–	–	–		1 (1)
Sum		12,033	112 (125)	36 (41)	10 (10)	20 (25)	5 (10)^a^	*183 (*211)

No dogs x-rayed: Number of radiographed dogs for each breed where lesions were found in the same period. New Reg.: Number of dogs of this breed registered in Norwegian Kennel Club the year before. Uneven bilat: Different lesions in collateral elbows

MOS, medial ossified structure; MLL, medial lucent lesion; FME, fragmented medial epicondyle; FE, flexor enthesopathy

^a^Uneven bilateral: a: MLL-MOS, b: MLL-FE, c: FME-MOS, d: FE-FME. Included in sum

Of the 28 dogs diagnosed with bilateral lesions (15.3% of affected dogs), only five dogs had lesions of different categories in the right and left elbows (Table 1).

**Fig. 1:**
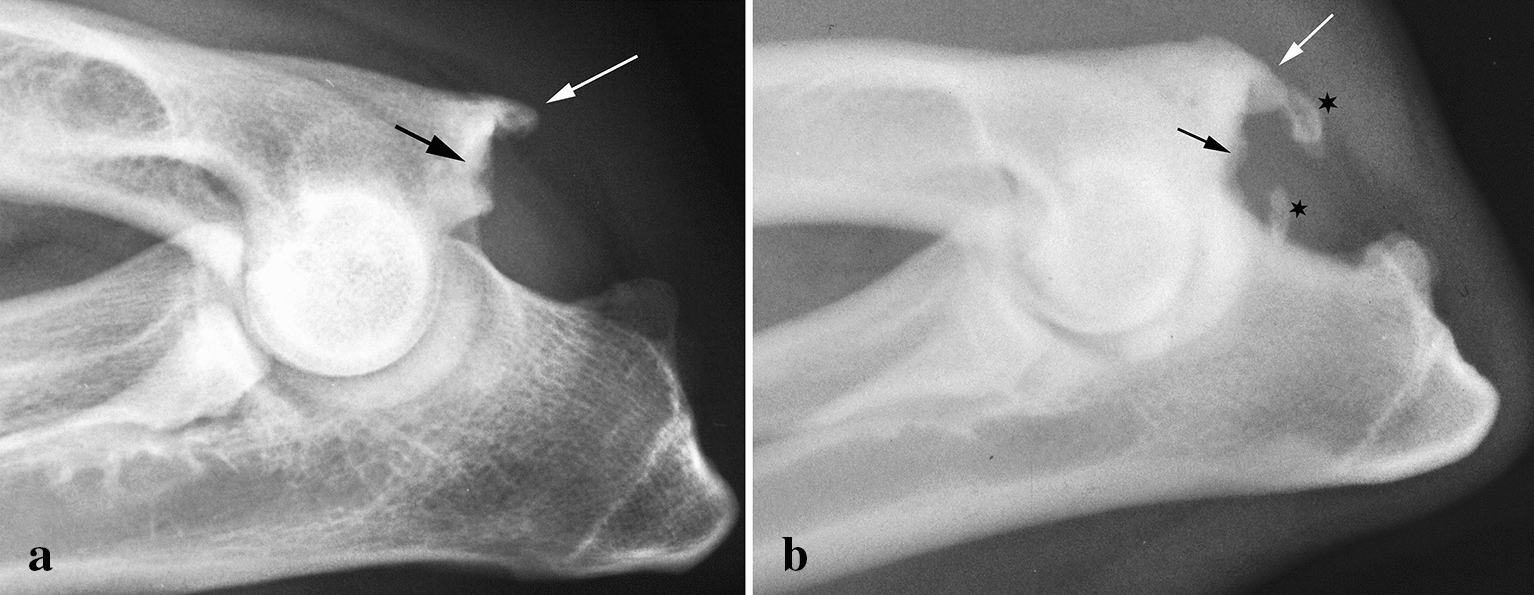
Flexor enthesopathy (FE) in mediolateral projections in an 18-month-old male Leonberger. **a** Note the bony spur formation on the distal-caudal end (white arrow) and the irregular, sclerotic structure in the bone distally in the medial epicondyle (black arrow). **b** Flexor enthesopathy (FE) in a 12-month-old female Labrador Retriever. Note the irregular bone distally (black arrow) and the bony spur distal-caudally in the medial epicondyle (white arrow). There are calcified structures (black star) distal to the medial epicondyle in the area of the carpal flexor tendons. There is also periarticular new bone formation in the joint and sclerosis in the semilunar notch indicating medial coronoid disease

FE (Fig. 1a, b) was found in 20 dogs and 25 elbows, representing 0.14% of the radiographed dogs, and three dogs had this lesion in one elbow and a lesion of another category in the other (2 being FME). All elbows diagnosed with FE had both bony spur formation and an irregular bone structure in the distal edge of the medial epicondyle. Calcified structures were found near the distal end of the medial epicondyle in 17 of the 28 elbows affected with FE (Fig. 1b) and no such calcification was found in the remaining 11 cases. Four of five bilateral cases had either calcification or no calcification in both elbows. There was no difference in mean age between dogs with FE and calcification and those with no calcification (26.6 months and 24.5 months, respectively). Ten dogs were females and ten were males. Thirteen of the 20 dogs were under 2 years old.

**Fig. 2:**
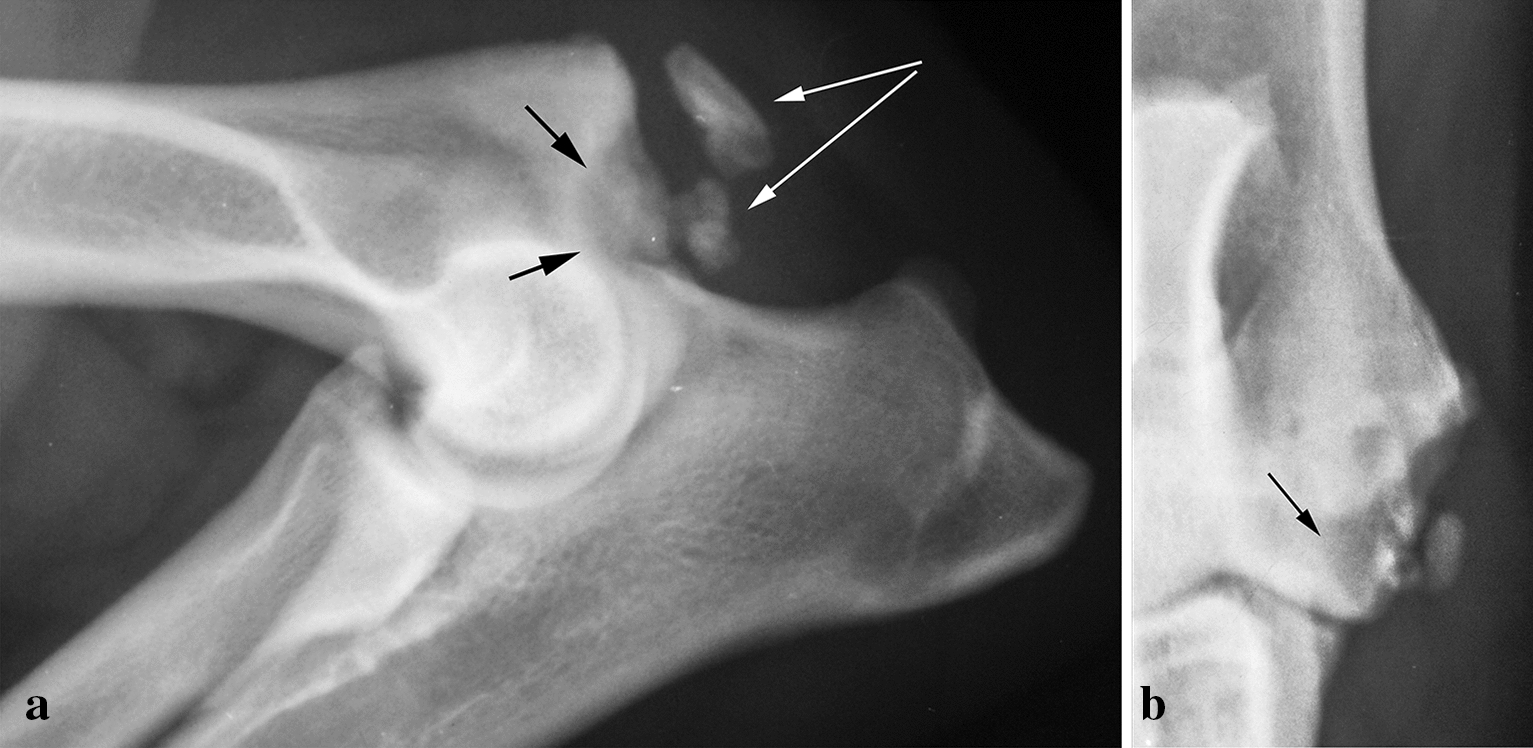
Fragmented medial epicondyle (FME) in **a** mediolateral and **b** craniocaudal projections in a 14-month-old male Rottweiler. Note the crater in the distal part of the medial epicondyle (black arrows) and the large bony structures (white arrows) visible in both views

FME (Fig. 2) was present in 10 dogs and 10 elbows, representing 0.07% of the radiographed dogs. In addition, three dogs had this lesion in one elbow and a lesion of another category in the other (two being FE). Eight were females and two were males, and eight of 10 dogs were under 2 years old.

**Fig. 3:**
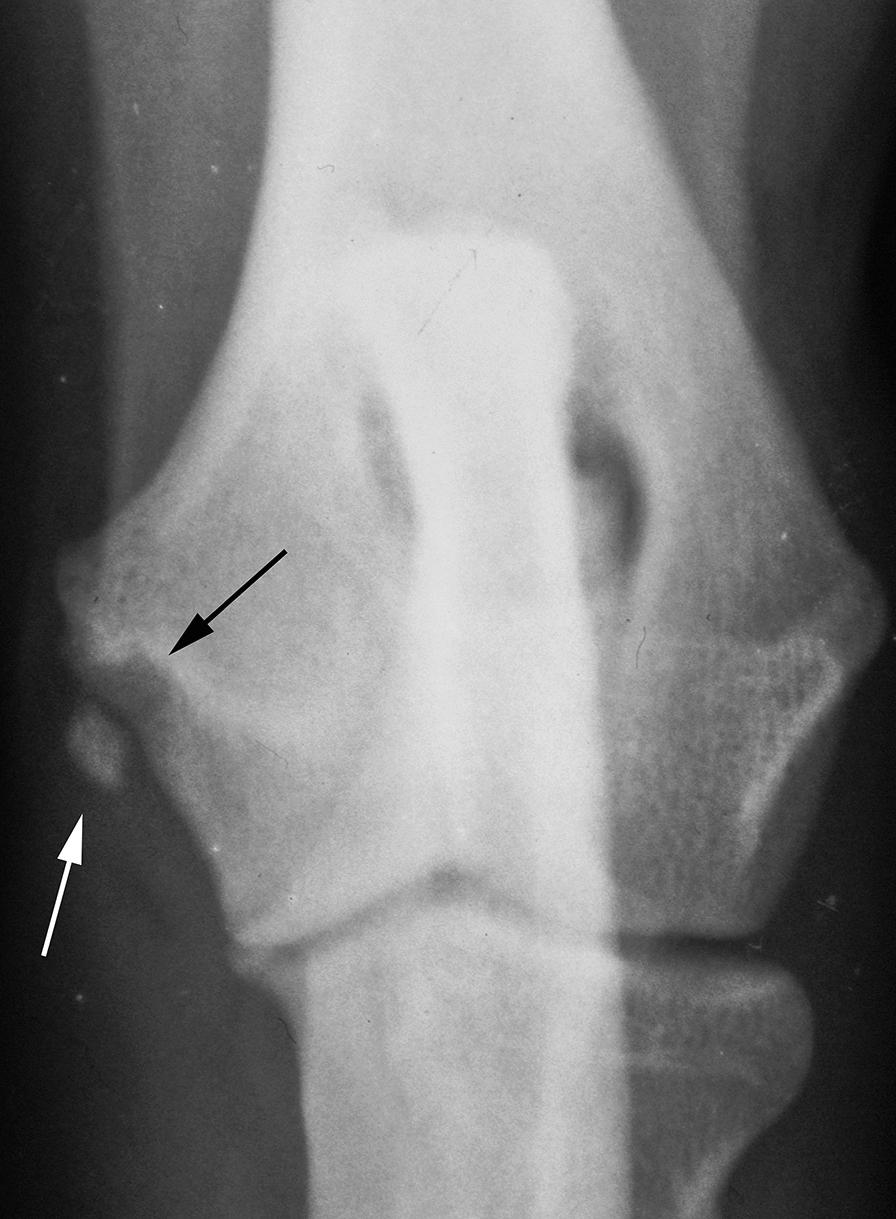
Medial lucent lesion (MLL) in a craniocaudal projection in a 19-month-old female Labrador Retriever. Calcified structure evident medially (white arrow). Note the lucent appearance in the adjacent bone (black arrow)

MLLs (Fig. 3) were found in 36 dogs and 41 elbows (0.25% of the radiographed dogs). Additionally, two dogs had MLL in one elbow and a lesion of another category in the other (one being MOS).

**Fig. 4:**
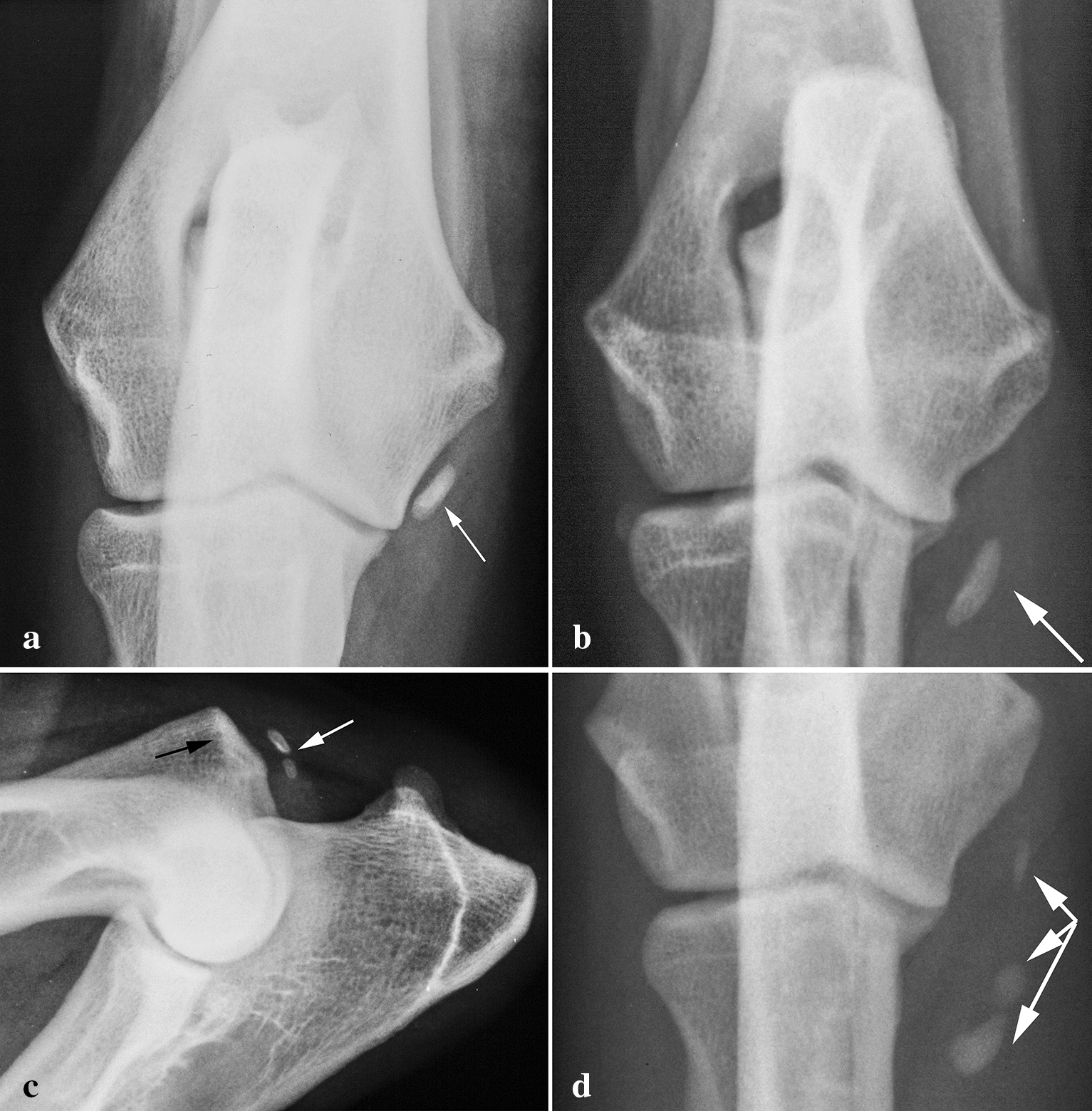
Medial ossified structure, in four elbows in craniocaudal in **a**–**c** mediolateral projections. **a** Medial ossified structure (MOS) in a middle location (white arrow) in a 12-month-old male Curly Coated Retriever. **b** MOS in a distal location (white arrow) in a 15-month-old female Howavart. **c** MOS in a caudal location (white arrow) in a 40-month-old male German Shepherd. Note the even structure of the distal edge of the medial epicondyle (black arrow) and the lack of spur formation and changes in the edge of the epicondyle. **d** MOS in various locations (white arrows) in the right elbow in a 34-month-old female Rottweiler. A similar lesion was present in left elbow

MOSs (Fig. 4a–d) were found in 112 dogs and 125 elbows representing 0.79% of the radiographed dogs. Additionally, two dogs had an MOS in one elbow and a lesion of another category in the other (one being MLL). The ossified structures were situated almost exclusively in two locations in the elbow: middle and distal.

The distribution of MOS locations is not provided in Table 1. Middle MOSs (Figs. 4a) were found in 63 dogs and 63 elbows, and distal MOSs (Fig. 4b) were found in 34 dogs and 39 elbows. Two dogs and three elbows had caudal MOSs (Fig. 4c) and MOSs were found in both the middle and distal locations in the same elbow in eight dogs and ten elbows MOS (mixed locations Fig. 4d). Middle MOSs were found at almost exactly the same spot in every case, while distal MOSs had a larger variation in location (varying distance to the joint space). Distal MOSs were generally larger (mean 33.2 mm^2^ ± 22.1 mm^2^) than middle MOSs (mean 18.4 mm^2^ ± 24.0 mm^2^). One Rottweiler in the present study was radiographed twice, and its middle MOS had increased in size between 6 and 16 months of age. There was, however, no difference in the size of MOSs in affected dogs older than 2 years compared to MOSs in those younger than 2 years.

### Breed differences

The prevalence of the various lesions differed between breeds (Tables 1, 2). Newfoundland dogs and Labrador Retrievers had a high prevalence of FE (0.33% and 1.04%, respectively). Both breeds also had a high incidence of MOSs, but in different locations, and many of them were bilateral. Newfoundland dogs had mainly distal MOSs, and very few were found in other locations. Labrador Retrievers had mainly middle MOSs. Of note, five of 10 MOSs in mixed locations were found in Rottweilers.

**Table 2:** The prevalence of medial ossified structures (MOS**)** in breeds with more than 500 radiographed dogs

Breeds	No. MOS/no. dogs
Newfoundland	22/574 (3.8%)
Labrador Retriever	24/1813 (1.3%)
Bernese Mountain dog	12/1147 (1.0%)
Rottweiler	12/1757 (0.7%)
Golden Retriever	4/702 (0.6%)
German Shepherd	21/3524 (0.6%)
Belgian Shepherd	0/584 (0)
Leonberger	0/628 (0)

### Periarticular new bone formation (PNBF)

In dogs affected with MOSs or MLLs, there was no difference in the occurrence or degree of PNBF between affected and unaffected elbows. This was also the case when older affected dogs were compared to young unaffected dogs. Elbows with FME and especially elbows with FE had a highly increased presence of PNBF compared to unaffected elbows (Table 3).

**Table 3:** Periarticular new bone formation (PNBF) in affected and non-affected elbows

	Total	No PNBF	With PNBF	% with PNBF
MLL	44	39	5	11.4
MOS	127	111	16	12.6
FE	28	5	23	82.1
FME	13	9	4	30,8
Sum	*212*	*164*	*48*	
Unaffected	*154*	*134*	*20*	*13.0*

MOS, medial ossified structure; MLL, medial lucent lesion; FME, fragmented medial epicondyle; FE, flexor enthesopathy

## Discussion

In total, 183 dogs and 211 elbows were found to have medial humeral epicondylar lesions or medial ossified structures and were further studied. FE was found in 0.14% of examined dogs and FME was found in 0.07% of examined dogs. The most common findings in the study were MOSs, found in 0.7% of the examined dogs. FE has a significantly higher prevalence in some breeds like Labrador Retrievers and Newfoundland dogs.

### Relevance of the material

This material is unique. It is a prospective, observational study describing whatever may appear in the elbows of more than 14,000 dogs radiographically evaluated for ED. Contrary to all previous studies but one, the material in this study is not significantly biased regarding clinical signs. There are strong procedures to avoid a first-line selection by radiographing veterinarians in Norway. The material is biased regarding age, size, and breeds, because mainly young dogs of large breeds are radiographed for ED.

Most puppies born in Norway are registered with the NKC, and a significant number of individuals from some specific breeds are frequently registered in both the NKC and radiographed for ED (Table 1). Comparing the number of new registrations of dogs from a particular breed and the number of radiographed dogs from this breed one year later (Table 1) gives an indication of the degree of participation in the ED screening program for this breed. All calculations and other evaluations of breed differences in this study was restricted to breeds where the number of radiographed dogs exceeded 500. This makes the screening material of elbow radiographs representative for the elbows of the true population of young individuals of these specific breeds. Thus, presented differences in the prevalence of various lesions between dog breeds in this study is probably real. A limitation of this study is the lack of clinical data for the included dogs.

### FE: a diagnosis or a group of lesions?

A very important aspect of this discussion is that the enthesis is a highly specific tissue often called ‘the enthesis organ’ [20] and enthesopathy is a specific change in this tissue. The use of the term ‘flexor enthesopathy’ indicates a known aetiology, as enthesopathy refers to a disorder involving the attachment of a tendon or ligament to bone. Conclusively, when there is no sign of pathological changes in entheses, but also when lesions are not proven to have their origin in the enthesis per se, and particularly lesions occurring nowhere near the enthesis, it should not be categorised as an enthesopathy. In the present study, FE was a specific diagnosis of a specific lesion. Radiographically, it was characterised by the presence of a ‘bony spur’ formation on the distal-caudal end of the epicondyle and/or irregular bone structure in the distal border of the epicondyle itself.

It might be mentioned that an argumentative description in the proposal was that all the histological reports of calcified bodies in the elbows show the same pattern with bone in the centre and cartilage and collagen tissue in the periphery. However, Meyer-Lindenberg et al. [8], who presented most of the histological examinations in the literature, reported that five of 13 bone structures surgically removed from the joints contained pure bone. In the review on medial humeral epicondylar lesions from 2012 it was stated that only one report described ‘bony spurs’ [15]. This might indicate that very few cases in the studied literature were cases of FE as originally defined [7]. Bony spurs have been found as an almost constant radiographic sign in elbows with FE in later studies [10, 11].

### FE diagnosed using radiography alone

A ‘bony spur’ is a popular term for enthesophytes in the enthesis organ [20], and thus, the most reliable indication of pathologic changes in the enthesis. One study, [10] found 80 elbows with primary or concomitant FE, using radiography and CT, and all elbows but two had developed a bony spur in the epicondyle. These two had an irregular bone structure at the distal edge of the medial epicondyle and could be diagnosed using radiography. The 120 elbows that did not suffer from FE had no changes in the medial epicondyle and could be excluded from the FE group using radiography alone. Furthermore, another study [11] reported that 11 of 13 elbows with primary FE had developed both bony spurs and irregular bone in the distal edge in the medial epicondyle. In one dog with bilateral FE, the only radiographic sign was a radiolucent area and sclerosis in the medial epicondyle itself. Bone lysis and sclerosis are other findings in enthesopathy. It has been concluded that radiography cannot be used to differentiate primary and concomitant FE [10, 12], and even that this differentiation cannot be done in all cases using CT [12, 14]. De Bakker et al. [12] reported that three dogs with primary FE had no clinical signs from the elbows and normal radiographic appearance.

It may be assumed that all 80 cases described by de Bakker et al. [11] and all cases described by van Ryssen et al. [11] were cases of ‘true’ FE and not some other lesion such as an MOS. In conclusion, if the medial epicondyle is clearly visible, radiography may distinguish FE from other lesions in most clinical cases, if not all. Conclusively, only a few cases of primary FE without clinical or radiographic signs will be missed in radiography.

### Exclusions and deficiencies in the material

The number of FE cases might be slightly underestimated in this study due to the inclusion and exclusion criteria. Radiographically visible PNBF found in a joint due to ED or another cause does not involve the most caudal part of the medial epicondyle until later stages. This is due to the location of the capsular attachment, where the primary PNBF is formed. The capsular attachment is situated axially and only halfway out on the medial epicondyle. Thus, PNBF will usually not be found caudally on the medial epicondyle when there is joint disease present and the typical spur formation of FE (which has nothing to do with PNBF) can be diagnosed. In some cases, however, the formation of PNBF in the form of periosteal reaction will spread caudally and reach the caudal end of the epicondyle. A spur cannot be differentiated with excessive formation of this periosteal reaction. Thus, elbows where PNBF exceeded the distal-caudal tip of the medial epicondyle in an excessive manner were excluded in the present study. Consequently, an unknown but small number of cases of ‘concomitant FE’ [10] might have failed to be included in our study. Other diagnostic modalities may have solved this problem. In addition, a few cases of primary FE might have failed to be included when the only radiographic sign was a radiolucent area in the medial epicondyle [11] as these findings were not described when the material was collected.

Most cases of FE described in the literature are from middle-aged dogs, and some are from older dogs [10–13]. The cause(s) and pathogenesis of FE are unknown. The dogs included in the present study were mainly young dogs with a mean age of less than 2 years. If ‘true’ FE is a secondary degenerative lesion, or for other reasons is developed mostly in older age, this study might have missed an unknown number of cases.

### Various lesions

FE has the radiographic characteristics of a degenerative type lesion. In the present study, however, FE was found in young dogs, and there was a quite striking difference in the prevalence of FE between breeds. This does not exclude that the pathological changes in the entheses are the result of degenerative development. It may, however, indicate that inherited factors for the development of these lesions are present, perhaps through faults in the structure of the soft tissue or the conformation of the elbow.

FE cases in this study cannot be differentiated as primary and concomitant. De Bakker et al. [10] introduced the expressions ‘primary FE’ and ‘concomitant FE’, and in 80 elbows with FE only, 12 were found to be primary. Considering these numbers and the incidence of osteoarthritis, the possibility of replacing the word ‘concomitant’ with the word ‘secondary’ has obviously been discussed by the group. However, further studies are needed.

Cases of FE presented as primary seem to be rare, and recent research indicates that this type might be even more exceptional. It has been suggested that even CT will not always distinguish between primary and concomitant cases [13], and de Bakker et al. [12] found that a significant percent of elbows presented as primary FE had subtrochlear bone sclerosis and an unclear delineation or abnormal shape of the medial coronoid process.

In the present study, all 28 elbows diagnosed with FE had what could be called the ‘typical’ bony spur at the distal-caudal part of the medial epicondyle and irregular bone structure at the distal edge of the medial epicondyle (Fig. 1a, b). Seventeen elbows had calcification seen as a band-like opacity along the distal end of the medial epicondyle in the flexed view and along the medial side of the elbow / ulna in the Cr-Cd view. The mean age of those with calcification and those without was similar. The 11 elbows with primary FE presented by van Ryssen et. al [11] had no mineralisation or bone formation in this soft tissue, other than the bony spur, although the mean age was 4 years. It is possible that calcification and bone formation will not develop in the soft tissue in cases with FE when not present from the initial stage.

MOSs were by far the most common lesion found in the present study. These were bony structures outside the joint in which the medial humeral epicondyle was essentially normal. There were no bony spurs or irregular edges in the epicondyle, as described when medial epicondylar lesions were present, and no radiolucent area and sclerosis in the medial epicondyle [10, 11]. If joint pain is present together with an MOS, it is almost impossible to determine whether the bony structure is causing the lameness or not. Mineralised objects found in an elbow with joint pain tend to be interpreted as the cause of the lameness, as when surgeons [22] removed the normal sesamoid bone [23] in the tendon of the supinator muscle in the elbows of eight lame dogs with good results. Although there are strong indications that MOSs are in fact of no clinical significance and should be regarded as an incidental finding, there is no way to demonstrate that on each individual patient. A strong indication of the lack of clinical importance is that MOSs do not create secondary changes in the elbows. There was no difference in the occurrence or degree of PNBF when joints affected with MOSs and unaffected elbows were compared (Table 3), and this was also the case when older, affected dogs were compared to young, unaffected dogs. Another indication was found in a follow-up study of seven litters of Labrador Retrievers (46 dogs) [14]. UMEs were diagnosed in seven dogs and eight elbows when Cr-Cd radiographs were studied. The authors made a remark that UME is not a proper name for the lesions found. The original radiographs presented in this study revealed that the medial epicondyle was essentially normal. The lesions found were identical to what is presented in our study as a middle MOS (Fig. 4a, b). Paster et al. [14] found that the degree of osteoarthritis and the clinical signs were equal in affected and non-effected elbows during the whole observation time, ending at the age of 6–8 years.

A 16-month-old dog included in our study from the ED screening program had been radiographed earlier in the Small Animal Clinic at the Norwegian University of Life Sciences. The ossified structure had increased in size between 6 and 16 months of age. This could be due to growth of the ossified structure, or a pre-formed cartilage object being increasingly ossified towards maturation, or both. In response, the size of MOSs in affected dogs younger than 2 years was compared to the size of MOSs in dogs older than 2 years. The size was not larger in the older dogs, indicating that these structures do not develop after 2 years of age. Ossification of a pre-formed cartilage object during maturation is the most probable pathogenesis.

FME is reported to result in lameness [1, 5, 6], and increased PNBF was found in these elbows in the present study (Table 3). It is unknown whether FME lesions regularly involve the joint capsule, but they do sometimes. Grøndalen and Braut [1] reported the surgical treatment and histology of a bony fragment removed from the medial side of the elbow in an English Setter. Later interpretation of the radiographs revealed that this was a case of FME. The fragment was partly incorporated in the joint capsule and had a surface of hyaline cartilage inside the joint. The other side of the fragment was intimately connected with the deep digital flexor tendon. A calcified structure (metaplasia) connected both to the joint capsule, and a tendon was reported by Meyer-Lindenberg et al. as well [8] in several cases when a metaplasia was surgically removed from the elbows. This connection between the capsule and the tendon might result in a painful stretching of the joint capsule during movement.

**Fig. 5:**
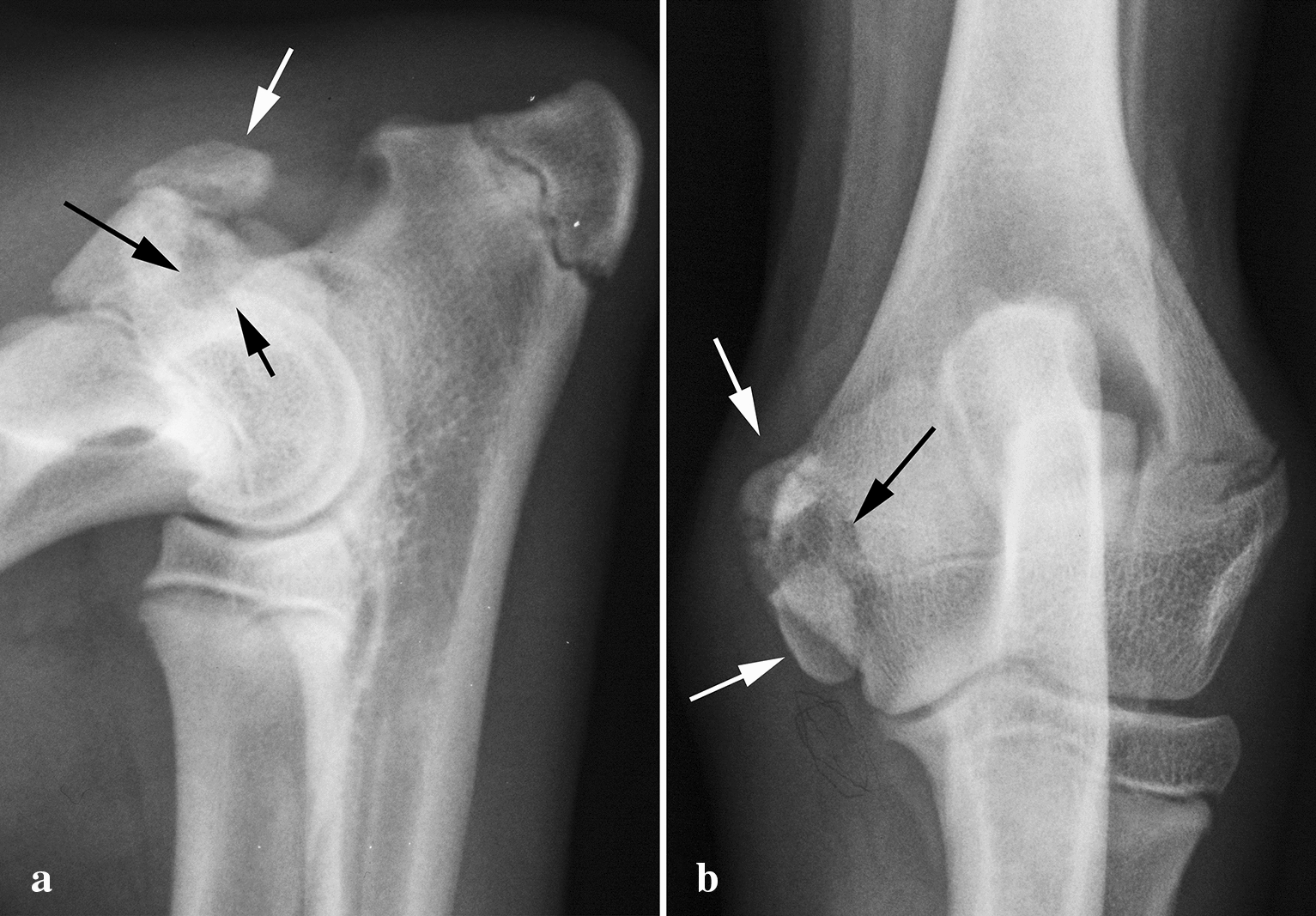
Fragmented medial epicondyle (FME) in **a** mediolateral and **b** craniocaudal projections in a 6-month-old male Wirehaired German Pointer. Note the large crater in the epicondyle (black arrow), the adjacent large bony fragments (white arrow), and that the edges of the centre of ossification are normal

Unlike FE, FMEs can present in puppies. The bony fragment(s) and the crater are visible in both projections, and in the young dog, the differentiation between FE and FME is simple. The radiographs of a 6-month-old German Pointer referred to the Small Animal Clinic at the Norwegian University of Life Sciences (Fig. 5a, b) reveal one such case with an extremely large crater and fragments. Careful interpretation reveals that this is not a UME, because a larger part of the centre of ossification is in normal position. It may be mentioned that these authors did receive some radiographs of young dogs with similar but more moderate radiographic appearance in the years when academia was the place to send these cases. The clinical signs were surprisingly insignificant but increased when cases were followed up until 4 years of age. Mahoney and Lamb [21] have also presented a similar case.

In the adult dog, FME is recognised mainly by a crater in the distal end of the medial epicondyle where the fragmentation took place (Fig. 2). The differentiation between FE and FME can be a challenge in adult dogs in cases where the fragments and the craters are smaller. As years go by, fibrous tissue, cartilage, and bone will proliferate and obscure the original findings. It would be strange if the entheses and the tendons were unaffected by this huge lesion in the area of tendon attachment. The caudodistal part of the medial epicondyle is normal at first, but a significant bony spur or enthesophyte in the tendon attachment may appear with increasing age and may, in some cases, be large. This development may be characterised as concomitant FE or rather secondary FE, and these cases might be misdiagnosed.

UMEs were not found in this study. In one case of FME, the fragment had grown. UMEs could have been suspected, but this diagnosis was excluded. The diagnosis of UME was introduced by Ljunggren et al. [5] and still is in the veterinary vocabulary. They presented one German Shepherd supposed to have this diagnosis, but the included radiograph revealed that the ossification centre was in the normal position. A later study on similar lesions stated that neither this presented case nor three similar cases the study presented [6] were UME. These authors postulated that UMEs do not occur and that the lesion was rather a fragmentation of the medial epicondyle, or in other words, a fragmented medial epicondyle (FME).

In the present study, it was impossible to analyse these lesions or to reveal their nature. They were included in this study because they appeared in the material and were relatively common in some breeds and not in others. There is a relatively large radiolucent area in the bone and a calcified structure in close connection or embedded in it, and the radiolucency seems not to disappear with age. Separately, they could probably be interpreted as some disturbance in the endochondral ossification in the epiphysis next to the physis. A similar radiolucent area was sometimes found without the presence of an ossified structure, but these cases were not included.

### Breed differences

Contrary to what was found in a prior study [15], breed differences are obvious in the present study (Tables 1, 2). The frequency of FE cases was found to be 1.4 in every 1000 dogs but much higher in specific breeds, such as Newfoundland dogs (10.4) and Labrador Retrievers (3.3), and much lower in others. None of the 3524 German Shepherds radiographed in this study were diagnosed with FE. Labrador Retrievers and Newfoundland dogs had a high prevalence of MOSs compared to many of the other breeds, and it was much higher if elbows instead of dogs were counted. In addition, while Labrador Retrievers had MOSs in all the various locations but mostly in the middle location, MOSs in Newfoundland dogs were almost exclusively found in the distal location (not differentiated in Table 1).

## Conclusions

It is critical to have more common terminology for lesions that are not FE and to name lesions. The descriptions of the lesions used in the present study may serve as a tool in further studies and discussion.

There are clear differences in the prevalence of lesions among the dog breeds documented in this study. The study shows that the prevalence of FE in a Norwegian population of mainly large dogs is approximately 1.4 in every 1000 dogs, but the number of cases of FE might be underestimated in this study. FE has a significantly higher prevalence in some breeds, such as Labrador Retrievers (3.3 in every 1000) and Newfoundland dogs (10.4 in every 1000). Some breeds, such as German Shepherds, are less affected. The prevalence of some of the other lesions such as MOSs and MLLs also varies according to breed (Table 2), and the prevalence of the location of MOSs varies significantly. The clear difference in prevalence between breeds might indicate that hereditary factors are present.

Specific breeds could be pointed out both as more and less susceptible to specific lesions. The presence of these lesions in the elbows, however, is not limited to a small group of dog breeds, as 28 breeds were affected, and mainly large dogs were investigated.

In our opinion, this study puts these lesions in a new perspective and reveals new information regarding the lesions. The presentation of this material, thus, may have added relevant questions and may enable discussions and be of help in further studies.

## Data Availability

The datasets used and/or analysed during the current study are available from the corresponding author on reasonable request.

## Abbreviations

ED: Elbow dysplasia
FE: Flexor enthesopathy
FME: Fragmented medial epicondyle
IEWG: International Elbow Working Group
MDC: Medial coronoid disease
MLL: Medial lucent lesion
MOS: Medial ossified structure
NKC: Norwegian Kennel Club
PNBF: Periarticular new bone formation
UME: Ununited medial epicondyle
